# Diversity of Gut Microbiota Metabolic Pathways in 10 Pairs of Chinese Infant Twins

**DOI:** 10.1371/journal.pone.0161627

**Published:** 2016-09-01

**Authors:** Shaoming Zhou, Ruihuan Xu, Fusheng He, Jiaxiu Zhou, Yan Wang, Jianli Zhou, Mingbang Wang, Wenhao Zhou

**Affiliations:** 1 Division of Gastroenterology, Shenzhen Children’s Hospital, Shenzhen, Guangdong, China; 2 Clinical Laboratory, Longgang Central Hospital of Shenzhen, Guangdong, China; 3 Shenzhen Following Precision Medical Research Institute, Shenzhen, Guangdong, China; 4 Division of psychology, Shenzhen Children’s Hospital, Shenzhen, Guangdong, China; 5 Shenzhen Imuno Biotech Co.,Ltd, Shenzhen, China; 6 Division of Neonatology, Children’s Hospital of Fudan University, Shanghai, China; 7 Key Laboratory of Birth Defects, Children’s Hospital of Fudan University, Shanghai, China; 8 Key Laboratory of Neonatal Diseases, Ministry of Health, Children's Hospital of Fudan University, Shanghai, China; Wageningen Universiteit, NETHERLANDS

## Abstract

Early colonization of gut microbiota in human gut is a complex process. It remains unclear when gut microbiota colonization occurs and how it proceeds. In order to study gut microbiota composition in human early life, the present study recruited 10 healthy pairs of twins, including five monozygotic (MZ) and five dizygotic (DZ) twin pairs, whose age ranged from 0 to 6 years old. 20 fecal samples from these twins were processed by shotgun metagenomic sequencing, and their averaged data outputs were generated as 2G per sample. We used MEGAN5 to perform taxonomic and functional annotation of the metagenomic data, and systematically analyzed those 20 samples, including Jaccard index similarity, principle component, clustering, and correlation analyses. Our findings indicated that within our study group: 1) MZ-twins share more microbes than DZ twins or non-twin pairs, 2) gut microbiota distribution is relatively stable at metabolic pathways level, 3) age represents the strongest factor that can account for variation in gut microbiota, and 4) a clear metabolic pathway shift can be observed, which speculatively occurs around the age of 1 year old. This research will serve as a base for future studies of gut microbiota-related disease research.

## Introduction

The gut micobiota plays an important role in human health. However, the early colonization of microbiota in the human gut is a complex process and remains largely unclear. It is assumed that the microbiota colonization begin as early as during the first trimester urinary tract infection. Aagaard *et al*. systematically studied the placentas microbiota composition from 320 subjects, and compared them to other human body site microbiota. They revealed that placenta harbors a unique microbiota composition, although they are similar to the human oral microbiota [1]. Delivery mode is another factor that contributory to shape newborns gut microbiota during birth. Dominguez-Bello *et al*. revealed that vaginally delivered infants acquired microbiota similar to their own mother's vaginal microbiota, and C-section infants harbored bacterial communities similar to mother's skin surface [2]. The gut microbiota stabilization or maturation is affected by feeding model as well [3]. Furthermore, numerous researchs have indicated that antibiotics play an essential role in altering the gut microbiota and exert long-lasting effects during later life. Early exposure to low-dose antibiotics may disrupt metabolic homeostasis in microbiota of mice and lead to obesity [4, 5]. Decreased diversity of microbiota early in life has been associated with compromised immune development. Cahenzli *et al*. found that a failure to establish a critical level of diversity in the gut microbiota of developing mice may result in a long-term increasing in IgE levels, and then predispose mice to immune-mediated disorders [6]. Co-twins have been used to study how human genetics affect the composition of gut microbiota. However, only a few of phenotypic characteristics, mainly referring to age, rather than host genetics have been evaluated. Tims *et al*. [7] used 16S rRNA gene microarray to study the gut microbiota composition of 40 adult monozygotic (MZ) twin pairs, half of which were discordant with body mass index (BMI). Their results revealed that MZ twins have more similar microbiotas compared with unrelated subjects, and some gut microbes give rise to the BMI differences between twin pairs. So far, nevertheless, these studies have been mainly restricted to adults group [8] or the use of 16S rRNA gene based profiling [9]. To understand the composition of human gut microbiota during early life and to evaluate the effects of host genetics, we performed shotgun metagenomic sequencing of 10 pairs of Chinese twins, who ranged in age from 5 months to 6 years old. We systematically compared the diversity of gut microbiota between intra- and inter-twin pairs, and evaluated correlations between human phenotypes and gut microbiota at both the strain and pathway levels. We found a change in the genes involved in microbial metabolism when comparing the infants below one year of age with infants older than one year. Additionally, we also observed a trend that gut microbiota composition might begin to stabilize after 1 year old, and these changes, or the differences between younger infants (0–1 year old) and older babies (1–6 years old) were correlated with several functional pathways. Although there are certain limitations in our study, for example, we did not conduct continuous sampling and did not perform long-term follow-up, the current findings conduced to facilitate future studies.

## Materials and Methods

### Fecal sample collection and DNA extraction

10 pairs of twins were recruited from Shenzhen Children’s Hospital (Shenzhen, China), written informed consents were provided by the children's parents during a routine pediatric physical examination. The protocol of this study was in accordance with the Declaration of Helsinki, and was approved by the Human Ethic Committee of Shenzhen Children’s Hospital. Fecal samples were collected and stored at −80°C prior to DNA extraction implemented by Imunobio Co. Ltd (Shenzhen, China). DNA was extracted from fecal samples using a StoolGen DNA kit (CWBiotech Co., Beijing, China).

### Library construction and Shotgun Metagenome Sequencing

DNA concentrations were determined using a Qubit dsDNA BR assay kit (Thermo Fisher, Foster City, CA, USA) with 2 μl samples of extracted DNA. The libraries (insert size 200–500 bp) were constructed with a TruSeq DNA Sample Preparation kit (Illumina, San Diego, CA, USA) and an automated SPRI works System (Beckman Coulter, San Jose, CA, USA) according to the manufacturer’s instructions. QC (quality control) of each library was carried out using an Agilent 2100 Bioanalyzer (Agilent Technologies, Santa Clara, CA, USA), Qubit dsDNA BR assay kit (Thermo Fisher, Foster City, CA, USA), KAPA qPCR MasterMix, and a Primer Premix alone kit (Kapa Biosystems, Woburn, MA, USA) according to the manufacturer’s instructions. Libraries that passed QC (>3 ng/μl) were sequenced using an Illumina Hiseq2500 sequencer (Illumina, San Diego, CA, USA) instrument with the paired-end 150-bp sequencing model based on 2G raw data output per sample.

### Taxonomic and functional annotation of shotgun metagenomic sequencing

First, we filtered out reads that had an adapter or that were of low quality. Second, all reads were aligned to the human reference genome Hg19 to filter out reads with possible human contamination. Third, the latest version of MEGAN [10] or MEGAN5 [11] with default parameters was applied to the taxonomic and functional analyses of the shotgun metagenome dataset. Finally, taxonomic profiling and gene function annotation results based on KEGG pathways [12] and eggNOG analyses [13] were generated for further analysis.

### Phylogenetic, principle component, clustering, and correlation analyses

The Jaccard index was used to calculate the similarity and diversity of sample sets, in which eggNOG functional data for the samples were used according to the following formula: Jaccard index (sample A, sample B) = (sample A ∩ sample B) / (sample A ∪ sample B) [14]. R and PERL scripts (S1 File) were used for Principal Component Analysis (PCA), clustering, and correlation analyses.

## Results and Discussion

### Study design and shotgun metagenomic sequencing statistics

In the present study, five monozygotic (MZ) and five dizygotic (DZ) pairs of twins ranging from 0 to 6 years old were recruited during routine pediatric physical examinations at Shenzhen Children’s Hospital in Southern China (see sample characteristics in Table 1). Fecal samples were collected, shotgun metagenomic sequencing was performed, and averaged 2G raw data, or 13 million (M) 150-bp paired-end reads were generated using an Illumina Hiseq2500 sequencer. Clean datasets were aligned with the human reference Hg19 genome sequence to filter out human-related contamination, and the human origin contamination rate averaged 0.23%, which is relatively low. As shown in S1 Fig, most samples reach saturation to call 65 tax branches via 8 M reads. Statistics for these sequences are shown in S1 Table. Systematic taxonomy and gene function analyses were performed using MEGAN5 [11] (S1 and S2 Tables).

**Table 1:** Sample characteristics of 10 pairs of co-twins.

Sample ID	Sample Another ID	Sex	Month	Height (cm)	Weight (kg)	Twins (DZ/MZ)
Twins-4A	A5	F	5	62	6.7	MZ
Twins-4B	A5	F	5	61	5.7	MZ
Twins-5A	A7	F	7	73	9.3	DZ
Twins-5B	A7	F	7	71.5	8	DZ
Twins-2A	A8	F	8	70.5	8.4	MZ
Twins-2B	A8	F	8	71	8.4	MZ
Twins-1A	A13	F	13	86	10.9	MZ
Twins-1B	A13	F	13	86	11.1	MZ
Twins-3A	A22	F	22	77.5	9.1	MZ
Twins-3B	A22	F	22	79	9.3	MZ
Twins-7A	A36	F	36	98.5	13.1	DZ
Twins-7B	A36	M	36	102	15.2	DZ
Twins-10A	A36	M	36	96.3	16.7	DZ
Twins-10B	A36	M	36	94.5	15.2	DZ
Twins-8A	A36	M	36	98.3	12.6	DZ
Twins-8B	A36	F	36	100.7	16.5	DZ
Twins-6A	A60	F	60	118.1	19.7	DZ
Twins-6B	A60	M	60	116.7	17.8	DZ
Twins-9A	A72	F	72	109.8	17.6	MZ
Twins-9B	A72	F	72	111.8	17.6	MZ

Note: F, female; M, male; MZ, monozygotic twin; DZ, dizygotic twin.

### MZ pairs of co-twins share more gut microbes than DZ pairs of co-twins or inter-twins

**Fig 1:**
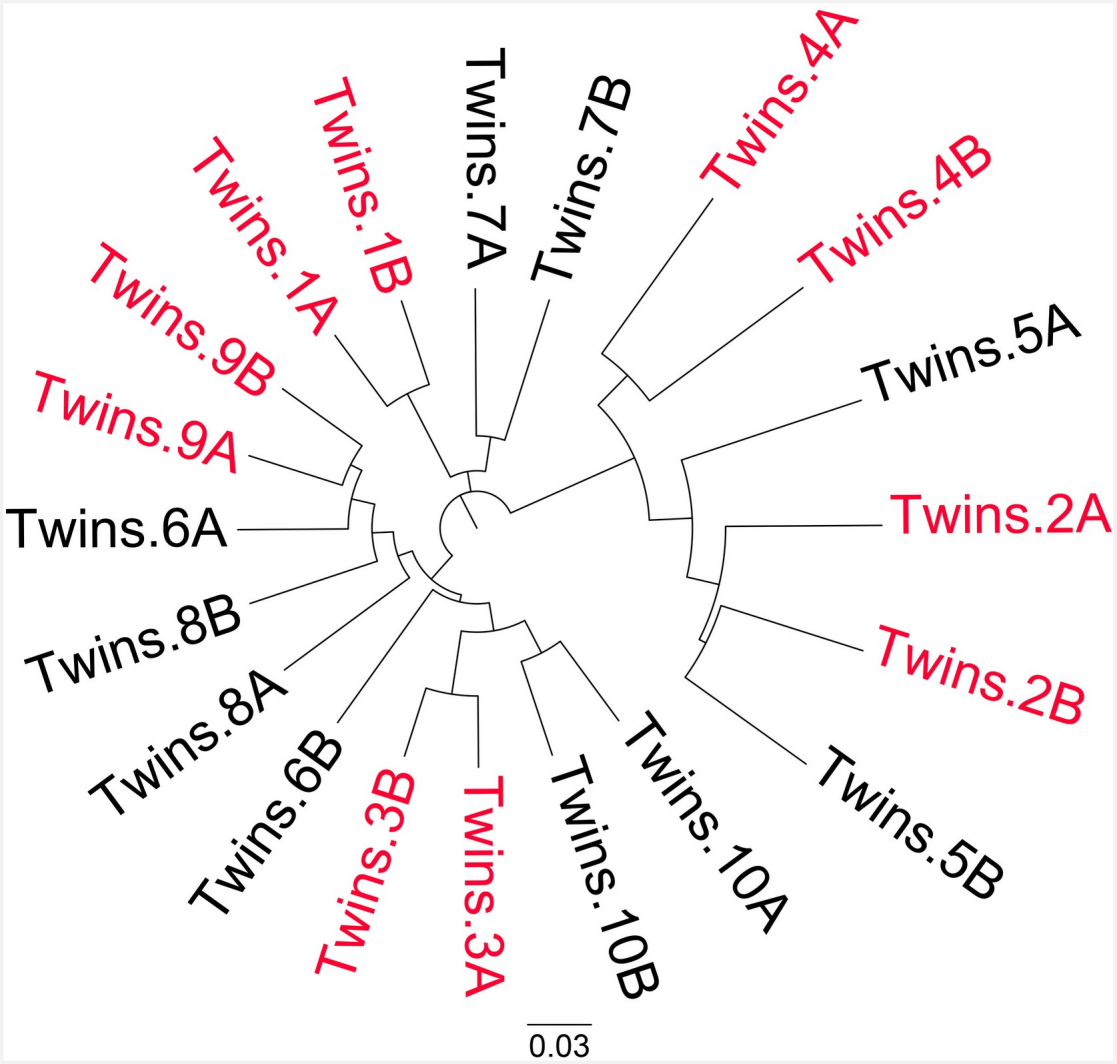
MZ co-twin pairs share more gut microbes than pairs of DZ co-twins or inter-twins. The sample distances between any two samples were computed using the 1–Jaccard index. MZ (monozygotic) and DZ (dizygotic) twins are marked with red and black font, respectively. This figure shows that compared with DZ and non-twins, MZ twins are more tightly clustered.

Previous metagenomic twin studies aimed at understanding differences in gut microbiota between intra- and inter-twins were limitedly performed in adults or used 16S rRNA gene based sequencing [8, 15, 16]. We applied the Jaccard index, an unweighted metric of community overlap [14], to measure the fraction of functional pathways that overlapped between intra- and inter-twin pairs (see Fig 1).

As shown in Fig 1, gut microbiota composition was similar between monozygotic and dizygotic twin pairs, and the similarity of gut microbiota was more attributable to genes or metabolic pathways than to strains, which was consistent with previous findings [15].

### Gut microbiota distribution is relatively stable at metabolic pathways level

**Fig 2:**
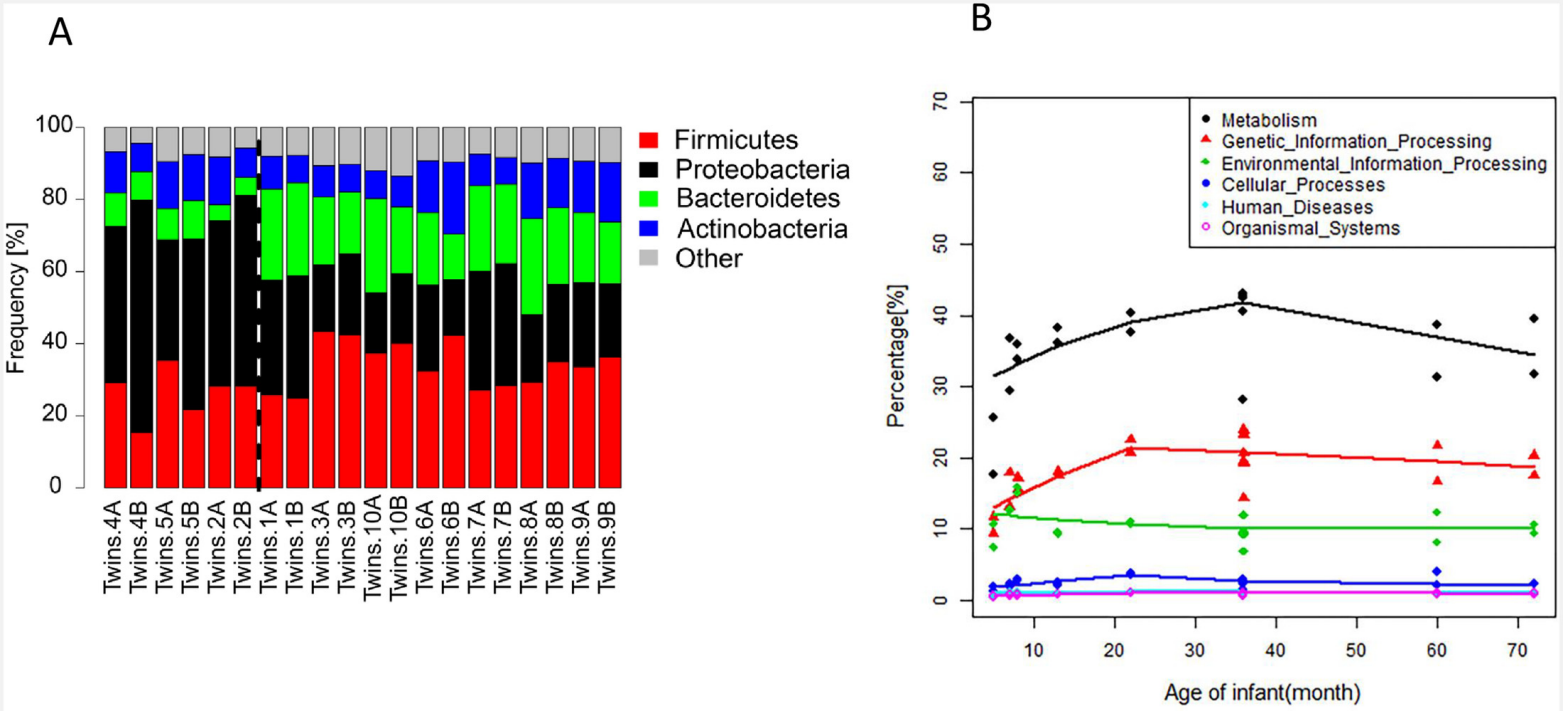
Gut microbiota are not stable and gut metabolism becomes stable with age. Fig 2a (top) is a stacked line of gut microbiota at the phylum level. The figures show that gut microbiota distribution are not stable at the taxonomic level. Fig 2b (lower) is a local fitting of gut microbiota at the KEGG level 1, the unique reads which are normalized to 1 million reads per sample annotated in each sectors are regressed against age (months) of 10 co-twins. The lines are drawn by R’s lowess according to a weighted polynomial regression method for the local fitting of KEGG level data. As the age increases, there is a trend that the KEGG functions for gut microbiota began to stabilize.

It is still elusive when gut microbiota stabilizes and which factor drives its maturation into an adult-like microbiota. Previous studies showed that gut microbiota is relatively more stable at the metabolic pathways level than at the taxonomic level [17]. In this study, we used MEGAN5 to draw radical, stacked line, and bar charts to demonstrate the gut microbiota distribution of 10 co-twins at the phylum level. Our results showed that the fluctuation of gut microbiota distribution at the phylum level was high, and this gut microbiota distribution at metabolic pathways level was relatively stable (Fig 2a and S8 Fig), which is consistent with previous results [17].

Early studies reported that gut microbiota is likely to mature into an adult-like microbiota by 1 year of age [9, 18, 19]. Using R’s lowess, a weighted polynomial regression method for local fitting, we generated simple bar chart based on different levels of KEGG data, and found that there was a trend that gut microbiota functional pathways began to stabilize at 1 year of age (Fig 2b), especially the genetic information processing and metabolism pathways. However, other functional pathways were stable over the whole period. This trend was becoming more obvious as height and weight increase (see S7 Fig), no other significant changes were observed for any other KEGG level (see S2 Fig).

### Age is the strongest factor that accounts for gut microbiota variation between samples

**Fig 3:**
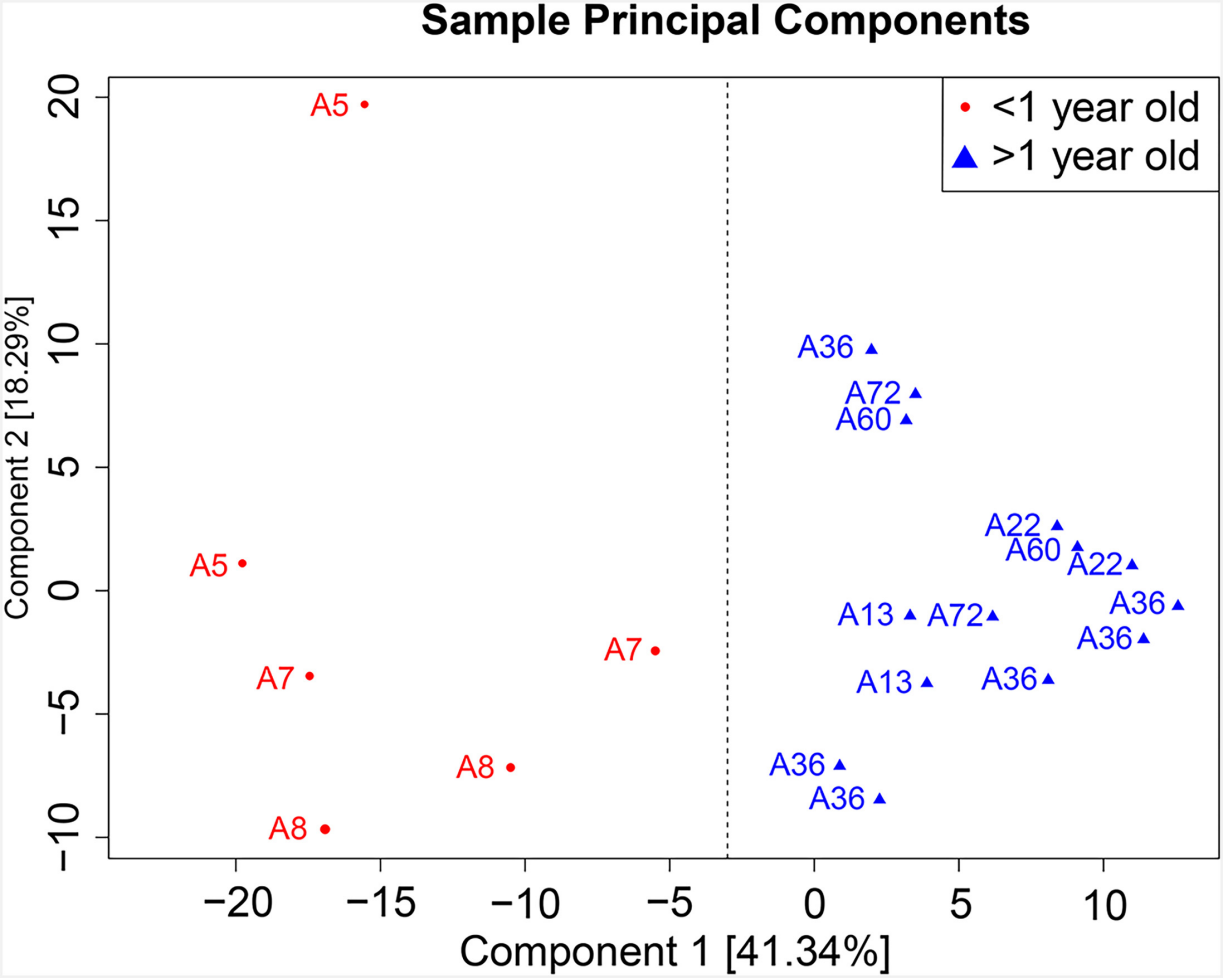
Age is the strongest component that affects gut microbiota composition at the KEGG pathway level. Samples were named using “A” plus infant ages according to months. Fig 3 indicates that the first and second dimension can account for 41.34% and 18.29% of the variation, respectively, and that the distribution of all samples in two dimensions and indicates that all samples could be divided into two groups based on age bifurcated at 1 year of age.

To identify factors that contributed to gut microbiota differences at the taxonomic and metabolic pathway levels, we carried out principal component analyses of all 20 shotgun sequenced samples according to the sample characteristics (such as age, weight, gender, or height), and found that age was the strongest driver in configuring infant gut microbial composition. Fig 3 revealed that the first and second dimension could account for 41.34% and 18.29% of the variation respectively, and that all samples in two dimensions could be divided into two groups based on age, using 1 year old as a cut-off value. Moreover, gender, height and weight were also important factors that affected the composition of gut microbiota (see S3 and S4 Figs).

It was apparent that these height and weight were closely related to age, thus, we carried out a correlation analysis between height or weight and age based on Pearson’s correlation coefficient. The results illustrated that age is strongly correlated with height and weight at the KEGG pathway level (data not shown). We subsequently identified pathways with correlation coefficients greater than 0.6, or less than –0.6 for further cluster analysis (Fig 3). It had been evidently observed that several pathways were significant enriched in younger (<1 years) and older (> 1year old) groups (see S9 Fig, S4 Table).

### Gut microbial diversity between younger and older twin pairs differ mostly in metabolic pathways level

It has been documented that gut microbiota probably stabilize over time in adults [17]; however, it remains obscure whether and when gut microbiota stabilizes in early life. Recently, Kostic *et al*. [18] reported that the gut microbial metabolic pathways, rather than taxonomies, remain stable during infancy. Whereas, a Swedish infant metagenome study by Backhed *et al*. [19] indicated that microbial metabolic pathways are not stable during the first year of life. They further reported that several functional pathway genes—the phosphotransferase system (PTS) genes, amino acid transporters genes, and B vitamin biosynthetic genes (e.g., vitamin B6, B7, and B9)—were enriched in newborns.

As we mentioned above, the changes in metabolic pathways were age-related, and the gut microbiota may begin to stabilize after 1 year of age. Therefore, we distributed samples into two groups bifurcated by 1 year of age and used Student’s *t*-test and nonparametric tests or the Wilcoxon rank sum test to identify significant differences in pathways between these two datasets. Considering that samples younger and older than 1 year of age were both clustered together, or showed balanced changes in functional profiling, few functional pathways showed a linear change with age.

**Fig 4:**
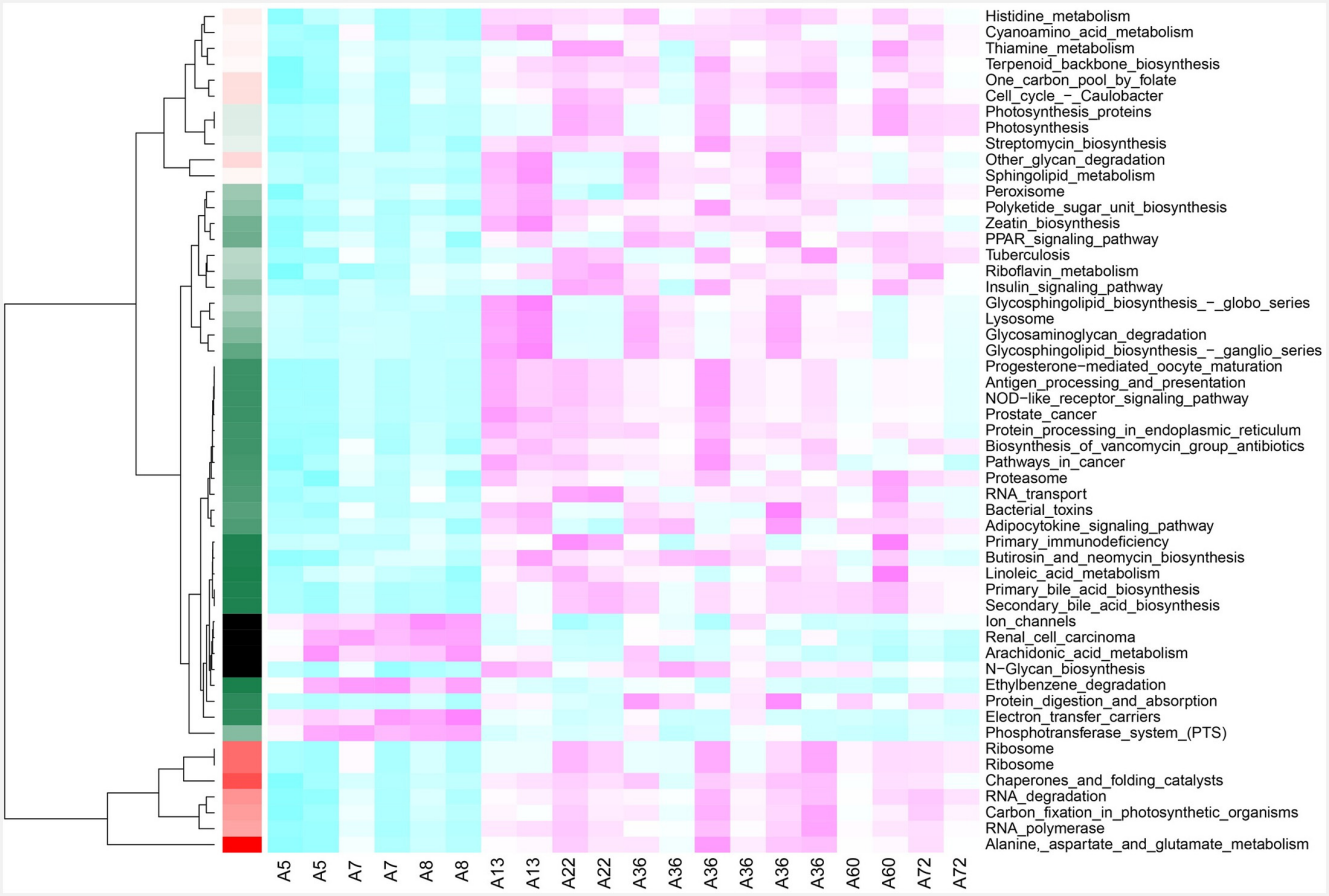
Revealing age-related KEGG pathways. Samples were renamed using “A” plus infant ages in months. The red color means these pathways are older age group enriched, the blue color means that these pathways are younger age group enriched. Significant, one year of age was used as the dividing line and samples were divided into two groups. All pathways with read count above 1000, a p-value less than 0.001, and a FDR value less than 0.05 were selected and clustered. The probability of several signaling pathways, such as renal cell carcinoma and arachidonic acid, occurring in the younger group is higher than for the older group.

We also used an independent sample *t*-test (Fig 4a) and found that the most significant pathways were distributed in areas with moderately or lowly reads counts of functional pathways. A similar situation could be observed in the analyses of other KEGG levels. Therefore, the impact of reads counts on functional pathways should be taken into consideration before analyzing, particularly on significant and well-characterized age-associated pathways. We mainly focused on pathways which reads counts of functional pathways were not less than 1/20 of the averaged reads counts of pathway items (1 M * 20 samples / pathway items). The p-values were calibrated according to the FDR (False Discovery Rate) and adjusted based on magnitude of the p-value. These significant age-related signaling pathways (Table 2) with reads counts not below 1000, a p-value less than 0.001, and a FDR value less than 0.05 were adopted in the heat map analysis (S10 Fig).

**Table 2:** Significant enriched pathways revealed by Student’s *t-*test in younger (<1 year old) and older (>1 year old) groups of babies.

FUNCTION ANNOTATION	YOUNGER AGE (<1YR) ENRICHED	ELDER AGE (>1YR) ENRICHED	ALL	T-TEST P-VALUE	T-TEST FDR	WILCOX TEST P-VALUE	WILCOX TEST FDR
Electron_transfer_carriers	6957	2731	9688	9.44E-04	4.83E-03	5.16E-05	4.90E-04
Primary_bile_acid_biosynthesis	422	5516	5938	1.66E-08	2.21E-06	5.16E-05	4.90E-04
Secondary_bile_acid_biosynthesis	421	5516	5937	1.56E-08	2.21E-06	5.16E-05	4.90E-04
Photosynthesis_proteins	8455	43333	51788	4.38E-06	1.16E-04	1.03E-04	6.38E-04
Photosynthesis	8454	43328	51782	4.39E-06	1.16E-04	1.03E-04	6.38E-04
Alanine,_aspartate_and_glutamate_metabolism	57162	223901	281063	3.94E-04	2.88E-03	1.03E-04	6.38E-04
Histidine_metabolism	13136	60692	73828	1.28E-05	2.63E-04	5.16E-05	4.90E-04
Cyanoamino_acid_metabolism	13299	53538	66837	6.38E-04	3.69E-03	3.61E-04	1.60E-03
N-Glycan_biosynthesis	304	1897	2201	7.51E-05	9.33E-04	2.06E-04	1.02E-03
Other_glycan_degradation	10208	83312	93520	1.72E-05	3.05E-04	5.16E-05	4.90E-04
Streptomycin_biosynthesis	9313	44280	53593	1.97E-05	3.27E-04	5.16E-05	4.90E-04
Polyketide_sugar_unit_biosynthesis	4652	27430	32082	8.64E-06	1.92E-04	5.16E-05	4.90E-04
Butirosin_and_neomycin_biosynthesis	939	4667	5606	2.00E-04	1.90E-03	2.06E-04	1.02E-03
Glycosaminoglycan_degradation	1679	27555	29233	8.23E-05	9.52E-04	5.16E-05	4.90E-04
Linoleic_acid_metabolism	614	3948	4562	6.92E-05	9.20E-04	2.06E-04	1.02E-03
Sphingolipid_metabolism	8321	59798	68119	2.27E-06	7.56E-05	5.16E-05	4.90E-04
Glycosphingolipid_biosynthesis_-_globo_series	3690	35283	38973	9.10E-05	9.73E-04	5.16E-05	4.90E-04
Glycosphingolipid_biosynthesis_-_ganglio_series	499	21163	21662	1.18E-04	1.20E-03	5.16E-05	4.90E-04
Ethylbenzene_degradation	2840	1931	4772	8.85E-04	4.83E-03	1.03E-04	6.38E-04
One_carbon_pool_by_folate	19436	70032	89469	5.16E-04	3.27E-03	5.16E-05	4.90E-04
Carbon_fixation_in_photosynthetic_organisms	30962	115161	146123	4.73E-04	3.14E-03	1.03E-04	6.38E-04
Thiamine_metabolism	15585	55039	70624	9.15E-05	9.73E-04	3.61E-04	1.60E-03
Riboflavin_metabolism	9088	32849	41937	6.06E-04	3.66E-03	5.16E-05	4.90E-04
Terpenoid_backbone_biosynthesis	15081	51297	66379	6.23E-04	3.68E-03	1.03E-04	6.38E-04
Zeatin_biosynthesis	5071	21024	26095	1.70E-05	3.05E-04	5.16E-05	4.90E-04
Biosynthesis_of_vancomycin_group_antibiotics	2252	10870	13122	3.93E-04	2.88E-03	1.03E-04	6.38E-04
Bacterial_toxins	4165	15371	19536	2.20E-04	2.00E-03	9.80E-04	3.07E-03
Phosphotransferase_system_(PTS)	17061	13293	30354	1.50E-04	1.48E-03	1.03E-04	6.38E-04
Ribosome	35929	151982	187911	9.35E-04	4.83E-03	9.80E-04	3.07E-03
Ribosome	35929	151982	187911	9.35E-04	4.83E-03	9.80E-04	3.07E-03
RNA_transport	3650	13802	17452	2.26E-04	2.00E-03	6.19E-04	2.39E-03
RNA_degradation	32468	121547	154015	4.39E-04	3.00E-03	5.16E-05	4.90E-04
RNA_polymerase	24176	113714	137889	6.90E-05	9.20E-04	5.16E-05	4.90E-04
Proteasome	2981	12355	15336	6.79E-04	3.84E-03	5.16E-05	4.90E-04
Chaperones_and_folding_catalysts	45964	166862	212826	6.82E-05	9.20E-04	5.16E-05	4.90E-04
PPAR_signaling_pathway	5257	19671	24928	5.18E-04	3.27E-03	2.06E-04	1.02E-03
Ion_channels	1443	1651	3095	3.31E-04	2.75E-03	1.03E-04	6.38E-04
Cell_cycle_-_Caulobacter	19309	69421	88731	3.71E-04	2.88E-03	1.03E-04	6.38E-04
Protein_processing_in_endoplasmic_reticulum	2177	11790	13968	3.86E-07	1.47E-05	5.16E-05	4.90E-04
Lysosome	1846	32268	34114	7.72E-05	9.33E-04	5.16E-05	4.90E-04
Antigen_processing_and_presentation	2010	10254	12264	2.47E-07	1.10E-05	5.16E-05	4.90E-04
NOD-like_receptor_signaling_pathway	2027	10265	12292	1.81E-07	1.10E-05	5.16E-05	4.90E-04
Insulin_signaling_pathway	6781	26692	33473	2.41E-04	2.07E-03	9.80E-04	3.07E-03
Progesterone-mediated_oocyte_maturation	2010	10254	12264	2.47E-07	1.10E-05	5.16E-05	4.90E-04
Adipocytokine_signaling_pathway	2831	13723	16553	5.42E-05	8.49E-04	6.19E-04	2.39E-03
Protein_digestion_and_absorption	710	8866	9576	4.81E-06	1.16E-04	1.03E-04	6.38E-04
Tuberculosis	8216	34137	42353	5.29E-04	3.27E-03	6.19E-04	2.39E-03
Pathways_in_cancer	3169	11083	14252	9.12E-04	4.83E-03	6.19E-04	2.39E-03
Renal_cell_carcinoma	1158	826	1984	4.17E-04	2.92E-03	6.19E-04	2.39E-03
Prostate_cancer	2010	10670	12681	1.99E-07	1.10E-05	5.16E-05	4.90E-04
Primary_immunodeficiency	1401	4672	6073	3.46E-04	2.79E-03	9.80E-04	3.07E-03

The probable signaling pathways, such as renal cell carcinoma and arachidonic acid, were detected more enriched in the younger group than that in the older group. By conducting a correlation analysis of infant phenotypes, such as age, height, and weight, as well as various levels of gut microbiota function, we found that several functional pathways were strongly link to age (Fig 4). Several functional metabolic pathways could be used to differentiate a younger infant from older baby twin pairs.

### Enrichment of several functional pathways in younger infants, including renal cell carcinoma and prion disease pathways

In accordance with the findings of Backhed *et al*. [19], the present study showed that functional pathway genes, including phosphotransferase system (PTS) pathway, amino acid metabolism pathway, cofactors and vitamin metabolism pathway, and carbohydrate digestion and absorption pathway genes, were enriched in infant microbiota prior to 1 year of age. Arachidonic acid metabolism pathway and ascorbate and aldarate metabolism pathway were abundant in in the younger infant group as well.

Although arachidonic acid, a polyunsaturated omega-6 fatty acid, is known to mediate gut inflammation [20, 21] and is associated with neurite outgrowth during early neuronal development [22], there is still a large knowledge gap in understanding the underlying mechanism. A recent study by Ardeshir *et al*. [23] found that the elevated levels of arachidonic acid in breast-fed *Rhesus macaques* may stimulate the production of TH17 cells and enhance immune function, then our findings could be important for understanding the relationship between diet and immune system development.

It has been reported that altered ascorbate and aldarate metabolism pathways are associated with a hepatocellular carcinoma phenotype [24]. A recent study by Suchodolski *et al*. found that ascorbate and aldarate metabolism was significantly (p<0.001) elevated in cats with diarrhea [25].

Our analysis may also reveal similar features of gut microbiota between early colonization and disease. Recent studies have shown that metabolites are involved in modulating immune function [26–28]. This finding indicates that similar metabolic pathways are used to establish and reactivate the immune system to some extent.

Interestingly, we also identified two human disease pathways—renal cell carcinoma and prion disease genes—that were enriched in the younger infant group. This finding has gone some way towards strengthen our data and broaden our understanding of shared metabolic pathways between early development and reactivation of the immune system. Nevertheless, further in-depth research would be important for elucidating the underlying mechanisms.

## Conclusion

In brief, by collecting infant twins and performing shotgun metagenome sequencing and systematic analysis, we found that twins share gut microbiota, which implicated that genetic factors contributing to gut microbiota composition. However, gut microbiota may also be strongly influenced by age, as attributing to differences in metabolic pathways, especially those bacterial groups involved in the genetic information processing and metabolism. There was a significant metabolic pathway shift observed in our study, including some novel metabolic pathways and others that have been associated with human disease, through comparing infants below 1 year of age to whom over 1year. Notwithstanding certain limitations (different time points come from different individuals), our findings will serve as a base for future research about gut microbe-related disease in infants.

## Supporting Information

S1 Fig Taxonomy rarefaction of 10 co-twins. (TIF)

S2 Fig Gut microbiota is shared between monozygotic and dizygotic pairs of twins. (TIF)

S3 Fig Bar plot of sample characteristics. (TIF)

S4 Fig Principle Component Analysis at the taxonomic and KEGG levels. (TIF)

S5 Fig Functional diversity between younger (<1 years old) and older (> 1 year old) group. (TIF)

S6 Fig Taxonomic diversity between younger (<1 years old) and older (> 1 year old) group. (TIF)

S7 Fig Local fitting for height and weight at functional level. A, height, B, weight.
(TIF)

S8 Fig A bar chart of gut microbiota at the phylum level. (TIF)

S9 Fig Pathways can differentiate younger from older groups. (TIF)

S10 Fig The distribution of read counts of pathways and p-values. (TIF)

S1 File R scripts for analysis. (ZIP)

S1 Table Statistics of shotgun metagenome sequencing results. (XLSX)

S2 Table Taxonomic profiling of 10 co-twins. (XLSX)

S3 Table Functional profiling of 10 co-twins. (XLSX)

S4 Table Significant enriched pathways in younger (<1 years) and older (>1 year old) groups. (XLSX)
