# Supplementary figures and images for: Diversity of Gut Microbiota Metabolic Pathways in 10 Pairs of Chinese Infant Twins
Source: PLoS One. 2016 Sep 1;11(9):e0161627. doi: 10.1371/journal.pone.0161627 (PMC5008625; doi:10.1371/journal.pone.0161627)

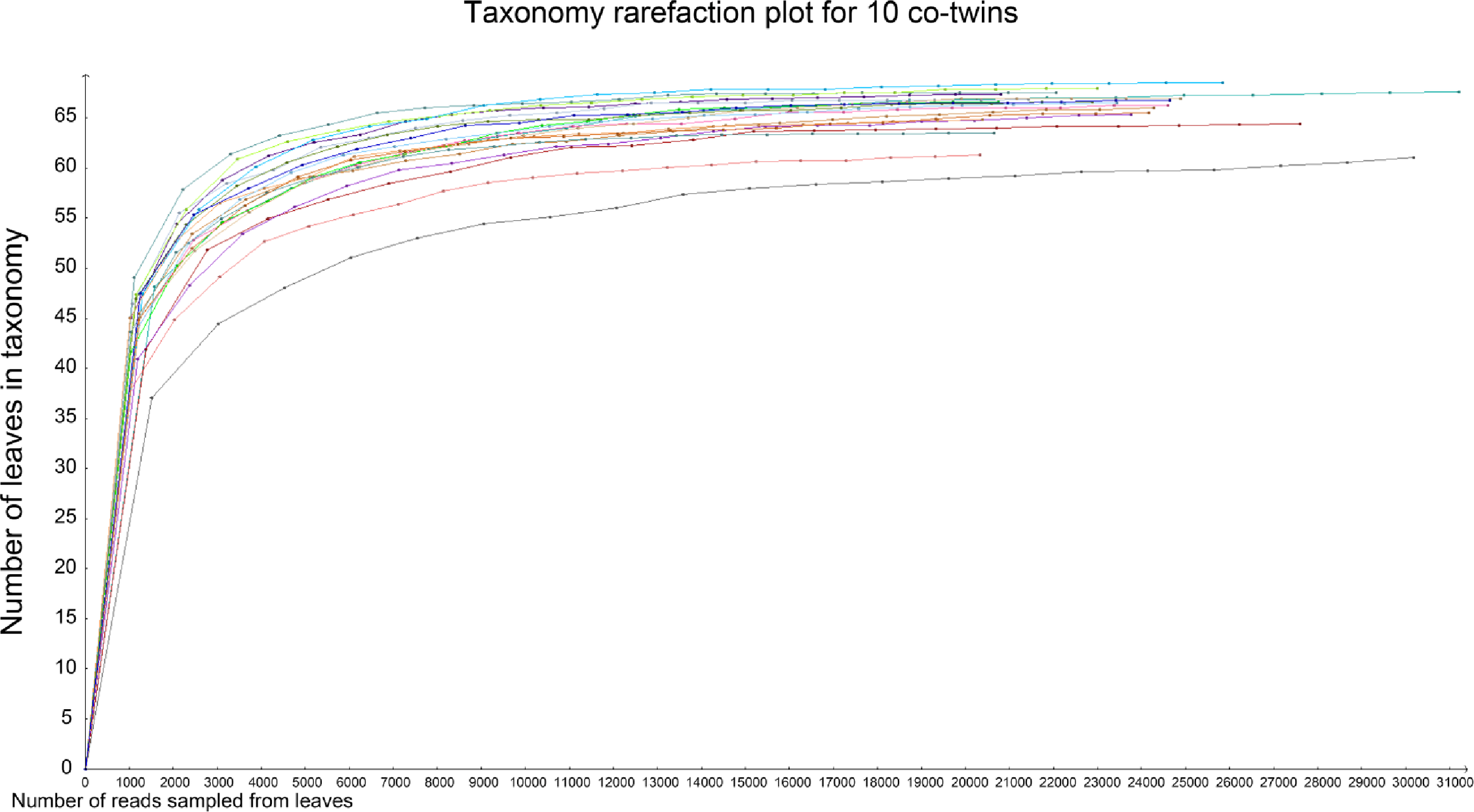

Supplement: S1 Fig — (TIF) [file pone.0161627.s001.tif]

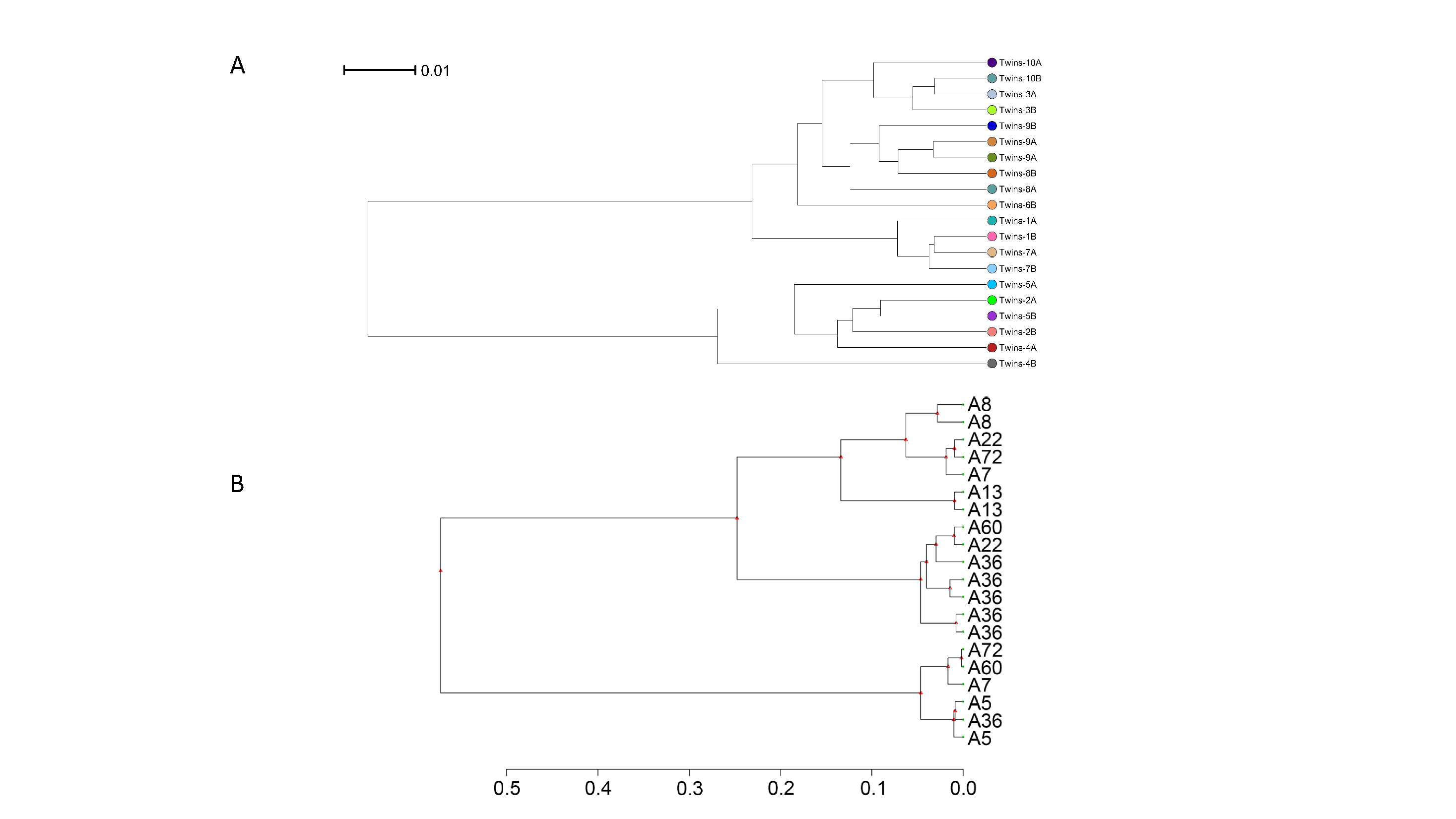

Supplement: S2 Fig — (TIF) [file pone.0161627.s002.tif]

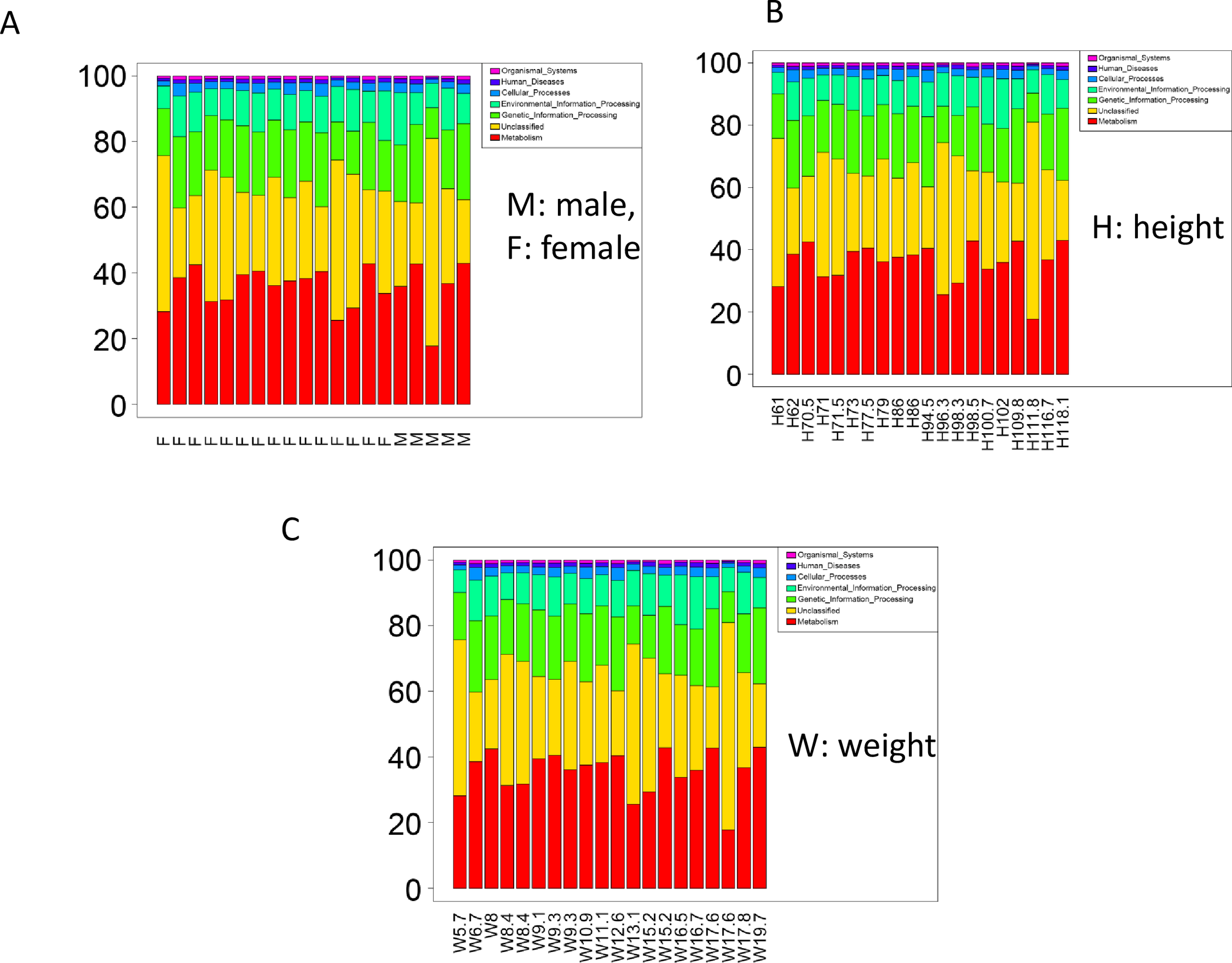

Supplement: S3 Fig — (TIF) [file pone.0161627.s003.tif]

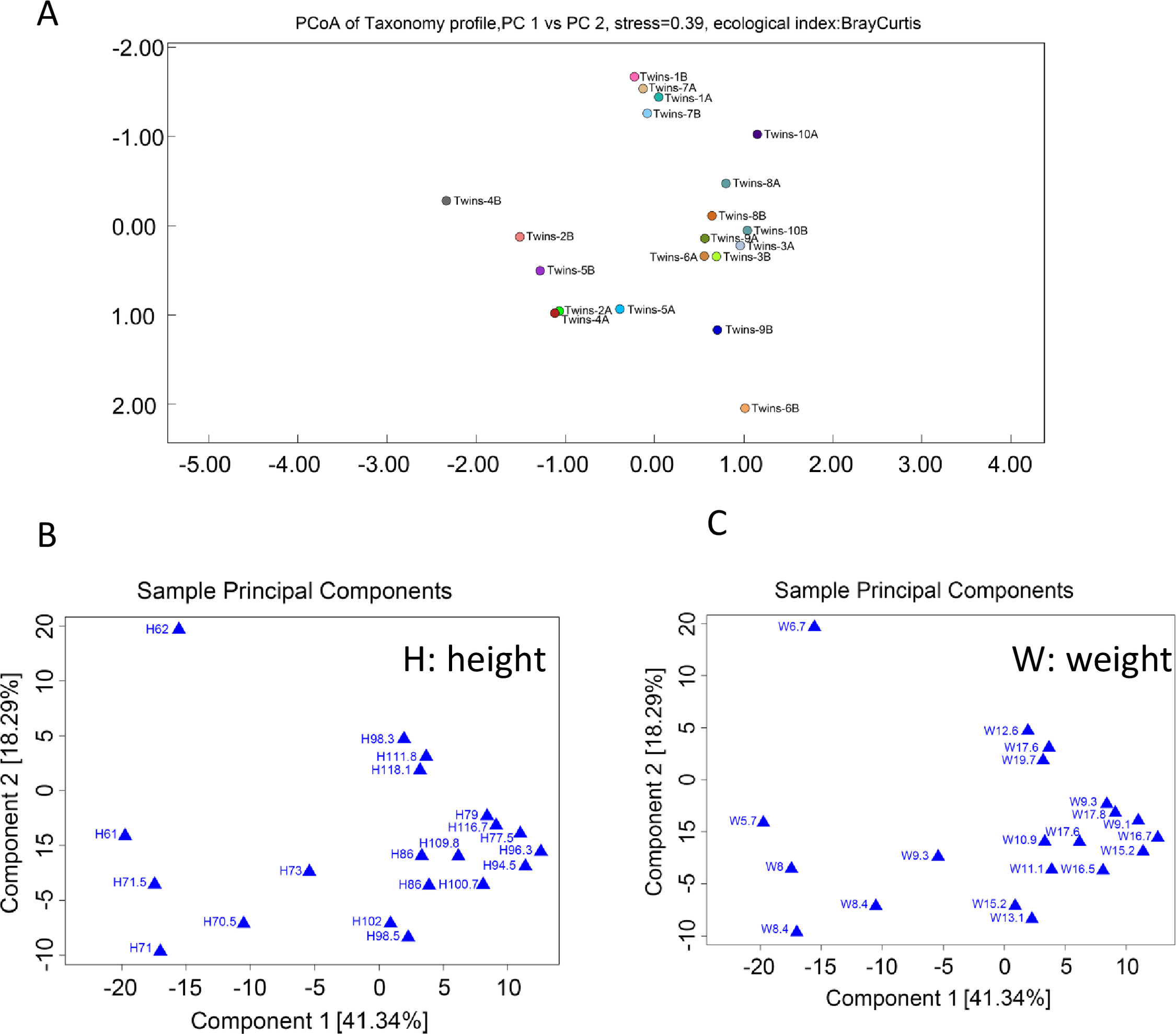

Supplement: S4 Fig — (TIF) [file pone.0161627.s004.tif]

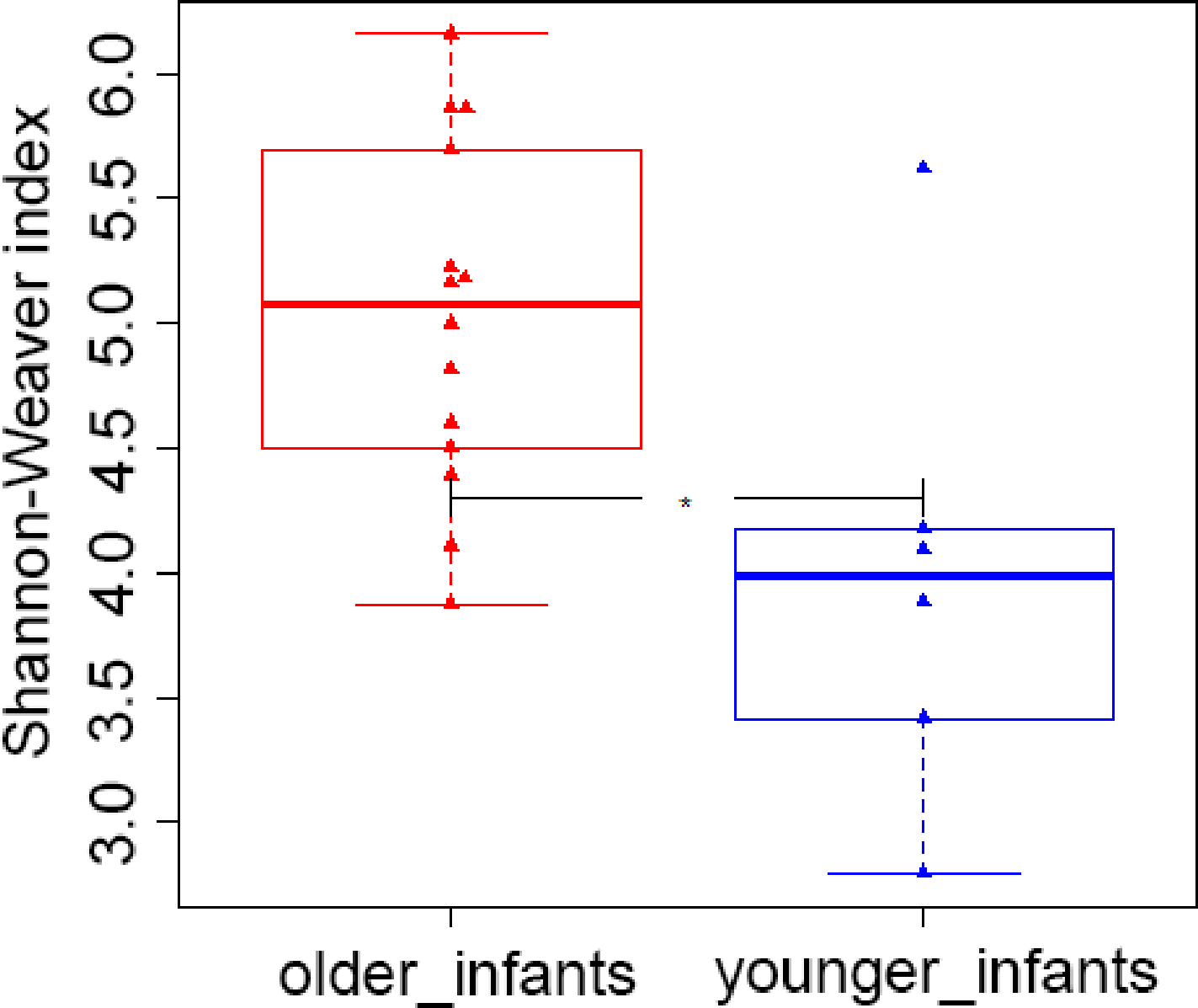

Supplement: S5 Fig — (TIF) [file pone.0161627.s005.tif]

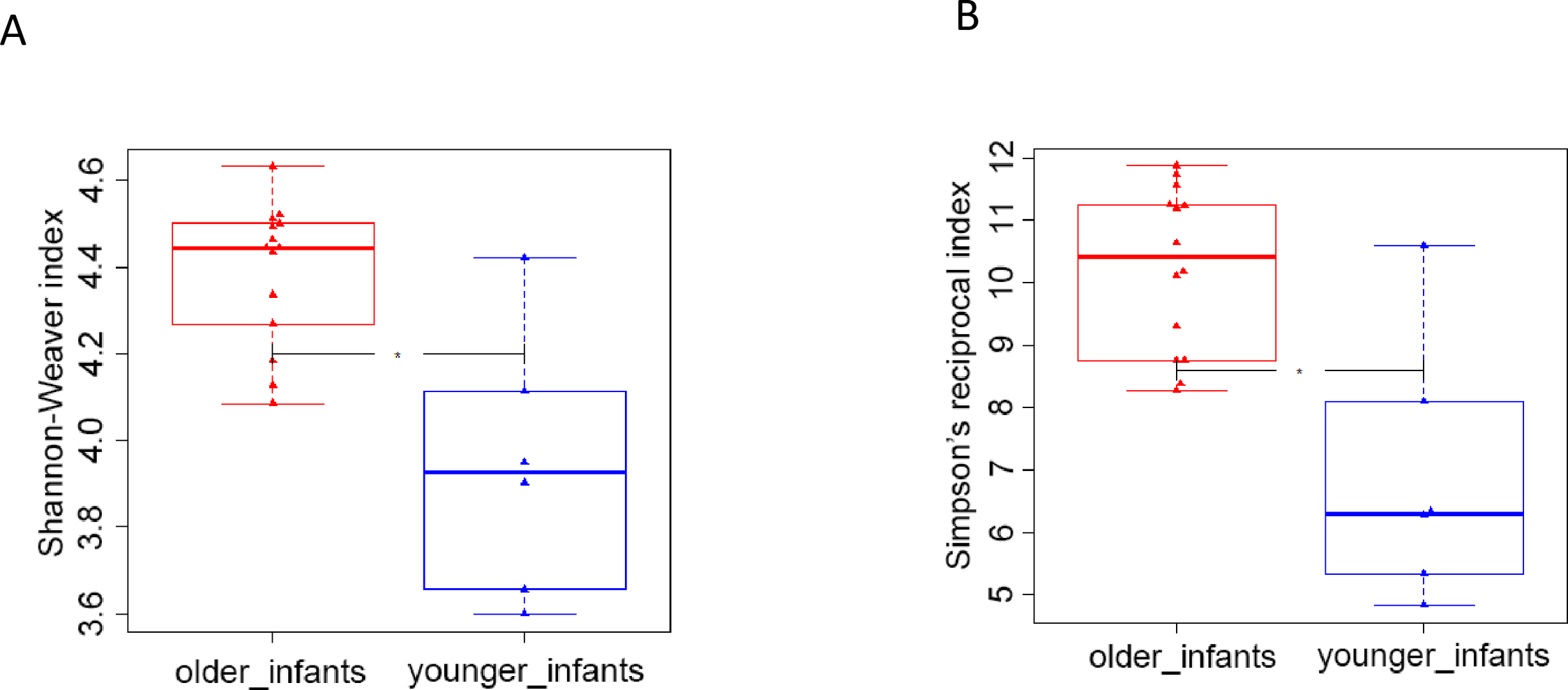

Supplement: S6 Fig — (TIF) [file pone.0161627.s006.tif]

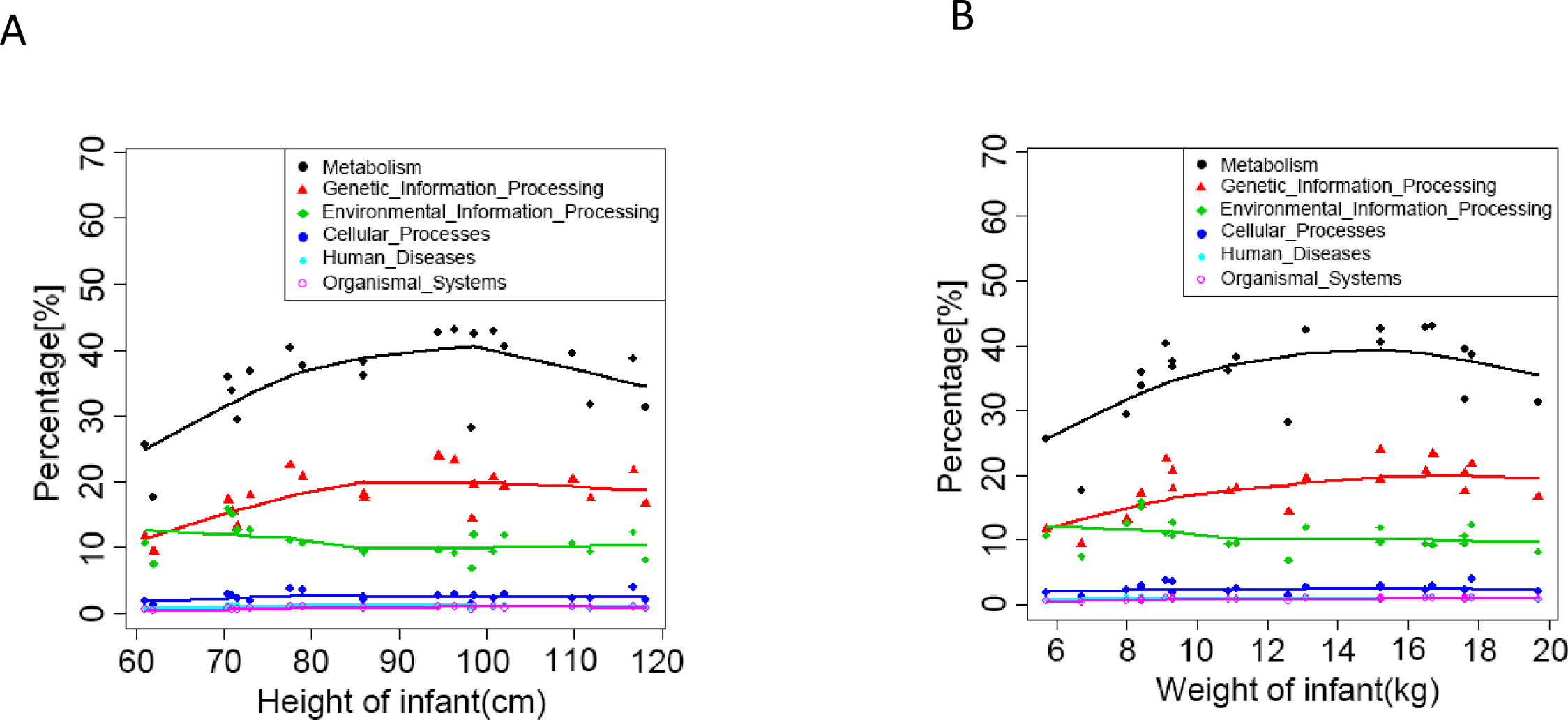

Supplement: S7 Fig — A, height, B, weight. (TIF) [file pone.0161627.s007.tif]

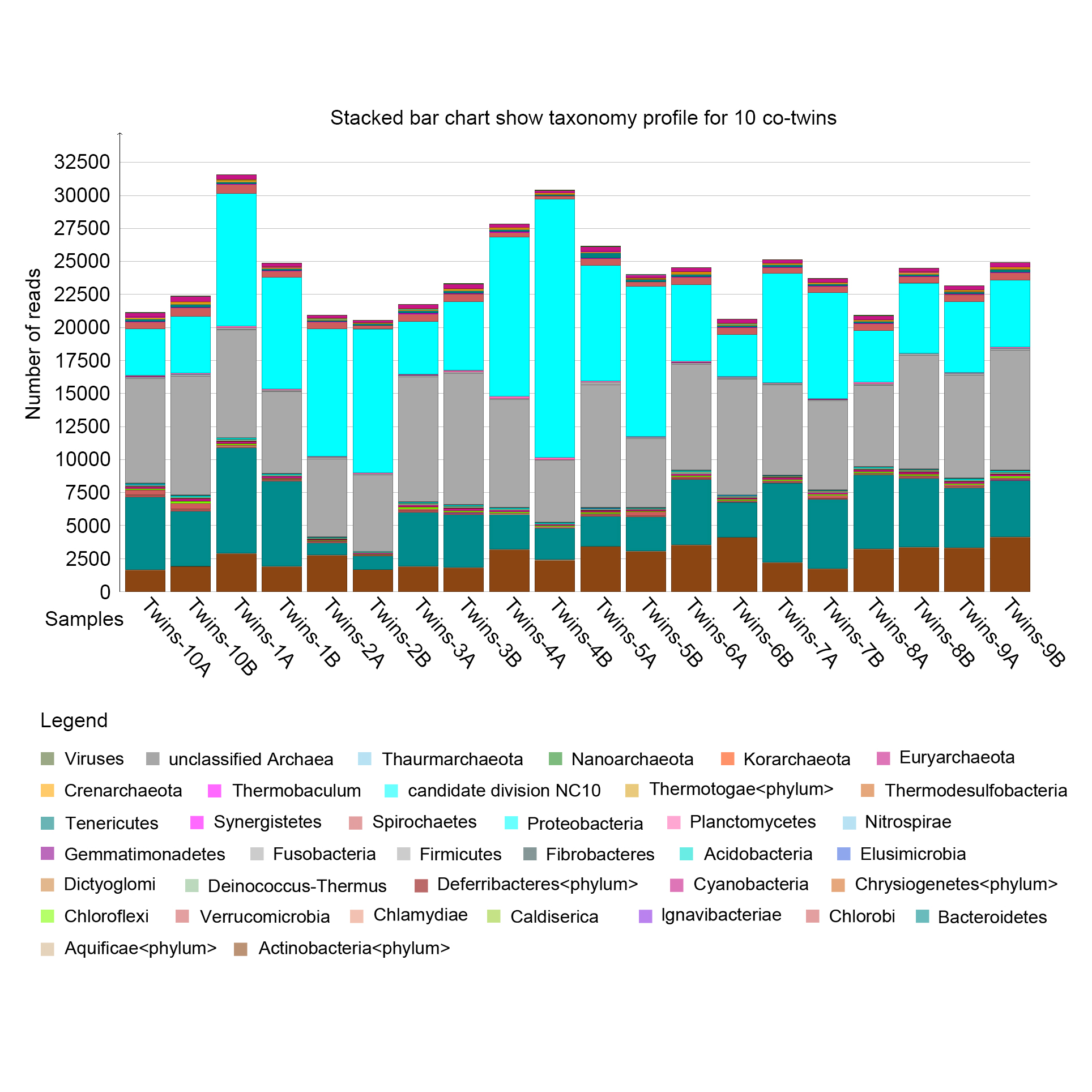

Supplement: S8 Fig — (TIF) [file pone.0161627.s008.tif]

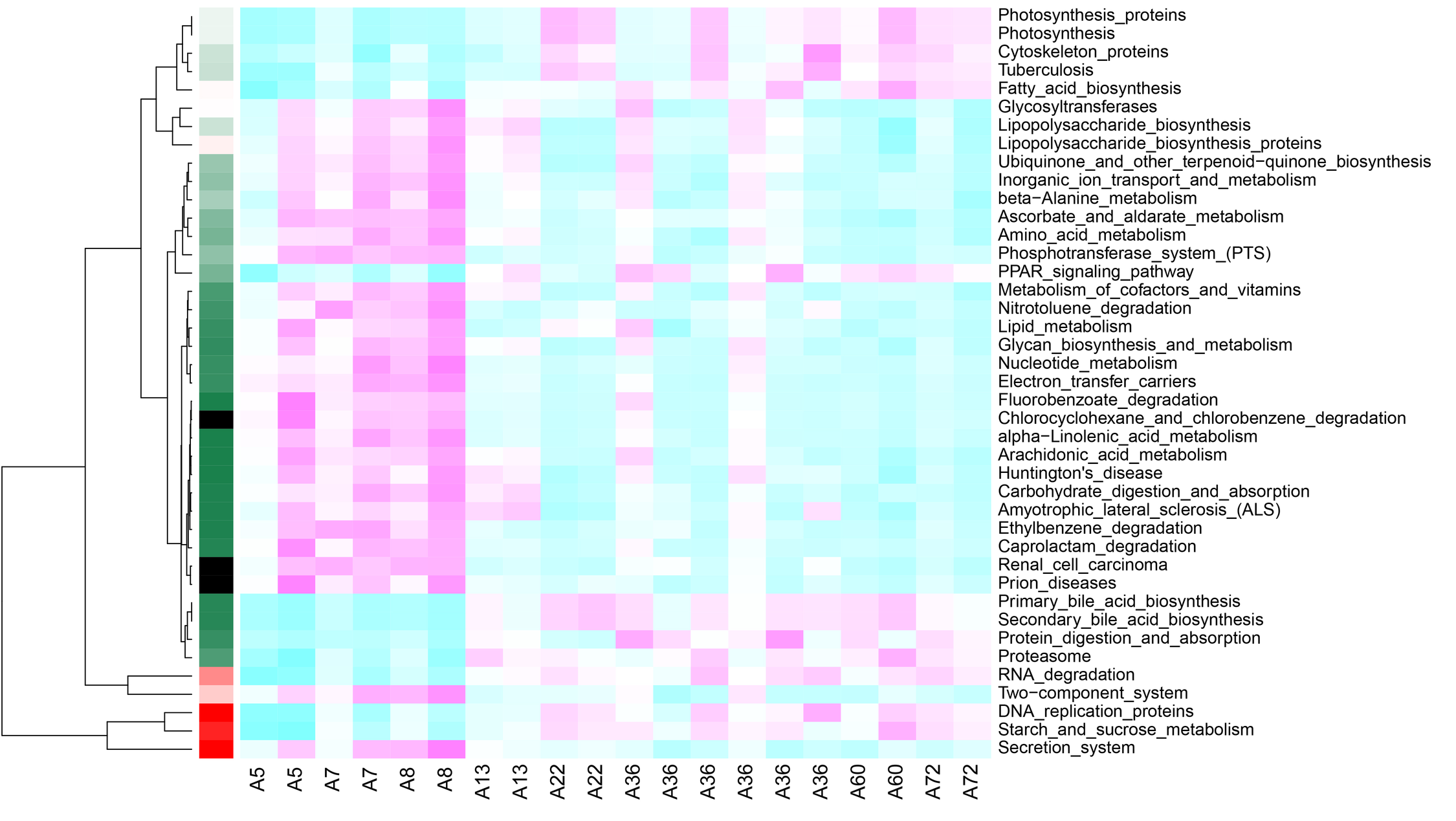

Supplement: S9 Fig — (TIF) [file pone.0161627.s009.tif]

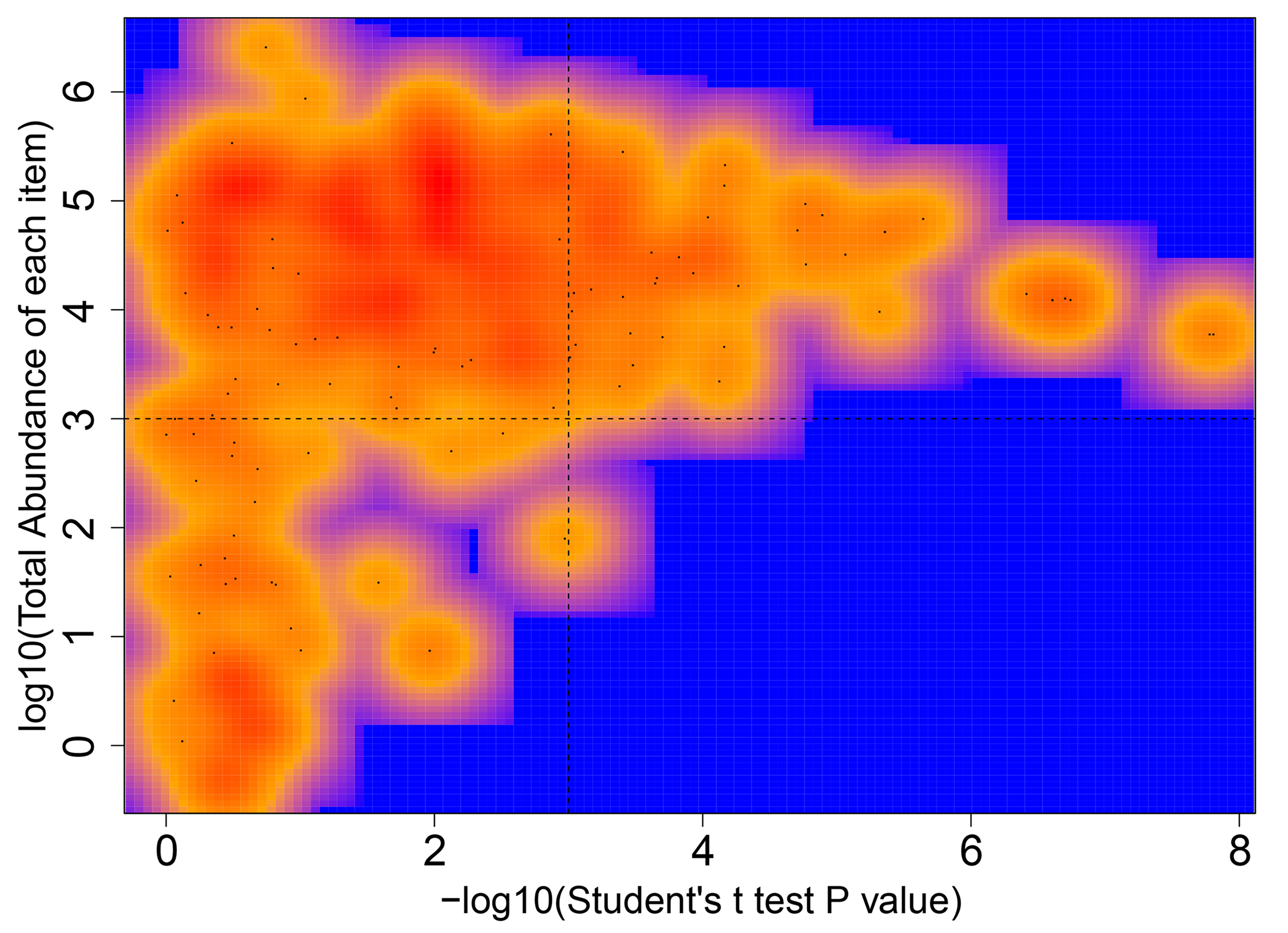

Supplement: S10 Fig — (TIF) [file pone.0161627.s010.tif]
